# Photoluminescence Lifetime Based Investigations of Linker Mediated Electronic Connectivity Between Substrate and Nanoparticle

**DOI:** 10.3389/fchem.2019.00207

**Published:** 2019-04-10

**Authors:** Jan F. Miethe, Franziska Lübkemann, Nadja C. Bigall, Dirk Dorfs

**Affiliations:** Institute of Physical Chemistry and Electrochemistry, Leibniz Universität Hannover, Hannover, Germany

**Keywords:** nanoparticles, photoluminescence, substrate, quenching, charge carrier dynamics

## Abstract

The evolution of systems based on nanoparticles as the main component seems to be a self-accelerating process during the last five decades. Hence, an overview across this field gets more and more challenging. It is sometimes rewarding to focus on the fundamental physical phenomenon of the electronic interconnection between the different building blocks of the obtained devices. Therefore, the investigation of charge transport among the utilized particles and their substrate is one of the mandatory steps in the development of semiconductor nanoparticle based devices like e.g., sensors and LEDs. The investigation of the influence of tunneling barriers on the properties of nanoparticle-functionalized surfaces is a challenging task. The different basic influences on the charge transport dynamics are often difficult to separate from each other. Non-invasive and easily viable experiments are still required to resolve the charge distributing mechanisms in the systems. In the presented work, we want to focus on thin and transparent indium tin oxide (ITO) layers covered glass slides since this substrate is frequently utilized in nanoelectronics. CdSe/CdS nanorods (NRs) are applied as an optically addressable probe for the electronic surface states of the conductive glass. The presented experimental design provides the proof of electronic interconnections in ITO coated glass/linker/NR electrodes via easy reproducible functionalization and polishing experiments. UV/Vis absorption and photoluminescence (PL) lifetime measurements revealed changes in the optical properties caused by differences in the charge carrier dynamics between the system. Our work is focused on the modification of charge carrier dynamics due to the application of linker molecules with different functional groups like (3-mercaptopropyl)methoxysilane (MPTMS) and (3-aminopropyl)trimethoxysilane (APTMS). The presented observations are explained with a simple kinetic model.

## Introduction

Indium tin oxide coated glass is one of the most common substrates in the field of nanoscience, since it is a transparent material with high electronic conductivity. The ITO coated glass slide is employed as typical nanoparticle carrying substrate in case of photoelectrochemical sensing applications (Wang and Wang, 2004a; Yue et al., 2013; Zhang, 2013), solar cells (Yaacobi-gross, 2011; Poppe et al., 2014), and screen technology. The application related improvement of this material is still ongoing (Savu and Joanni, 2006; Taniguchi et al., 2011). ITO is the composition of two oxides with the classical stoichiometric distribution of (In_2_O_3_)_0.9_) · (SnO_2_)_0.1_. ITO is chemically relatively inert and needs to be activated and functionalized in order to be integrated in devices (Kern and Puotinen, 1970). The functionalization of distinct surfaces like titania, silica, indium tin oxide (ITO), iron oxide, silicon and copper with silanes via silanization is a widely known and implemented process. Such modifications allow to obtain chemical surface characteristics, which are far away from the ones of the pristine substrates (Zhang et al., 2004; Ballarin et al., 2008; Yang D. et al., 2011; Lin et al., 2012; Liu et al., 2013; Poppe et al., 2013). Two of the most applied reagents to achieve the functionalization of surfaces are the silanes (3-aminopropyl)trimethoxysilane (Kern and Puotinen, 1970; Zhang, 2013) and (3-mercaptopropyl)methoxysilane (Doron et al., 1995; Miethe et al., 2018). This molecules are not only enabling the fixation of metal and semiconductor nanoparticles but also the implementation of novel technical processes like inkjet printing (Pavlovic et al., 2003; Xiao et al., 2005; Lübkemann et al., 2017). It is obvious, that the main difference of the mentioned linkers is their functional group. Thiol groups (MPTMS) are more acidic than their alcohol equivalents and form in this perspective a counterpart to amine groups (APTMS), which are the classical basic building block in organic chemistry (Cao et al., 2013). The nucleophilic character of deprotonated thiols leads to the chemisorption of subvalent and metal rich surfaces of semiconductor nanoparticles. Another factor is the sulfur affinity of metals like gold, which can be linked well to thiols (Zhang et al., 2004; Ballarin et al., 2008; Pensa et al., 2012). APTMS is widely known for its linking capabilities of metal nanoparticles. (Wang and Wang, 2004b; Zhang, 2013) The silanes 11-mercaptoundecyltrimethoxysilane (11-thiol) and *N*^1^-(3-trimethoxysilylpropyl)diethylenetriamine (3-amine) were applied as reference systems with longer carbon chains. The utilization of these molecules allows to investigate the influence of the carbon chain length on the probability of electron tunneling. However, a lower expected electronic interaction might be the reason, why these longer chained linkers are rarely utilized. Nonetheless, both molecules are suitable reference systems, since they show in principle the same chemical linking behavior to the ITO substrate and to the nanoparticles as their shorter chained analogous.

In the presented work a simple and easily producible ITO coated glass/linker/nanoparticle system was chosen out of the manifold of possibilities provided by literature (Hickey et al., 2000; Zhang et al., 2004; Chen et al., 2014; Miethe et al., 2018). PL lifetime measurements are utilized to reveal the electronic interconnection between the ITO and the semiconductor nanoparticles, which are linked to it via the above mentioned linker molecules. The stepwise preparation of the slides is based on (i) activation of ITO, (ii) functionalization of ITO, (iii) linking of nanoparticles to ITO, and (iv) finally polishing one of the two sides (either glass side or ITO side) of the substrate. To understand the electronic properties of an ITO/linker/nanoparticle electrode the optical properties of the system after every step of preparation were investigated and compared. Apparently, the results gained by our scientific study could not be obtained under consideration of solely the final system. Since the silanization of the soda lime glass side and the ITO side of the substrates was always realized simultaneously and thereby under the same conditions, the comparison of the functionalization process of both substrate surfaces was possible in case of all four linkers (short and long chained amino and thiol silanes). This allows the investigation of the influence of parameters like functional group and chain length of the linker molecule on the charge carrier dynamics. Furthermore, the effect of the surface modification of the particle as well as the electronic linking of the particle to ITO can be separated. A key point is to consider the polishing of one side of the slide, even when it is not an interesting chemical reaction, as a main step in the preparation process. Polishing excludes 50% of the nanoparticles in the system. This leads to the effective separation of effects from the linker itself and those caused by the linking to a conductive surface.

In detail the ITO coated glass/linker/nanoparticle system is based on one dimensional CdSe/CdS nanorods, which are known from the work of Carbone et al. (2007). This material is the result of a two-step synthesis. In the first step CdSe cores are obtained and in a second step applied as seeds to grow a rod shaped CdS shell around them. Accordingly, the direct semiconductor heterostructure shows a band structure, which is known as a pseudo-type II or type I in dependence of the core size (Eshet et al., 2013). CdSe/CdS nanorods were considered as a suitable material for the experiment since the PL quantum yield (QY) of the particles is high enough to guarantee results in an acceptable experimental timescale of minutes. Furthermore, the radiative lifetime of the particles is commonly much higher than in other direct semiconductor nanoparticles without heterostructure (Crooker et al., 2003; Tessier et al., 2012; Eshet et al., 2013). Another benefit of the chosen nanoparticles is their high surface per particle in comparison to dots of the same atom number. The latter fact might facilitate the particle linking to the substrate. The PL wavelength of the chosen nanoparticles is in the orange/red regime and thus far away from the wavelength of the excitation beam of 420 nm. There is no reported fluorescence of bulk ITO, as applied in our case, in contrast to ITO nanoparticles (Ma et al., 1995; Yang H. et al., 2011; Liang and Liu, 2018).

Overall it was possible to show a significant difference in the contribution of thiol silanes (MPTMS, 11-thiol) and amine silanes (APTMS and 3-amine) to the electronic interconnection of nanoparticles to their substrates. This effect can be related to electronic states caused by chemisorption of thiol or amine groups to the nanoparticle surface.

## Experimental Section

### Materials

Tin doped indium oxide coated unpolished soda lime float glass with a surface resistance of 12 ohms/sq (ITO slides) and 1.1 mm thickness were purchased from VisionTek. Cadmium oxide (CdO, 99.99%) and selenium (Se, 99.99%) were acquired from Alfa Aesar. Hexylphosphonic acid (HPA, ≥ 99%) and octadecylphosphonic acid (ODPA, ≥ 99%) were purchased from PCI synthesis. Toluene (99,7%), ammonium hydroxide (NH_4_OH, 28–30%), hydrogen peroxide (H_2_O_2_, 30%), sulfur (99.98%), (3-aminopropyl)trimethoxysilane (APTMS, 97%), (3-mercaptopropyl)trimethoxysilane (MPTMS, 95%) and *N*^1^-(3-trimethoxysilylpropyl)diethylenetriamine (3-amine, technical) were bought from Sigma-Aldrich. 11-mercaptoundecyltrimethoxysilane (11-thiol, 95%) was purchased from Gelest Inc. Tri-*n*-octylphosphine oxide (TOPO, 99%) and tri-*n*-octylphosphine (TOP, 97%) were obtained from abcr. 2-Propanol was purchased from Carl Roth.

### Instruments

UV/Vis absorption spectra of the CdSe/CdS nanorod solutions and the nanoparticle decorated substrates were measured with an Agilent Cary 5000 absorption spectrophotometer equipped with an Agilent DRA-2500 integrating sphere in center mount position. Emission spectra and the PL lifetime of the solution and the prepared ITO samples were recorded with a Fluoromax-4 spectrometer from Horiba equipped with a time correlated single photon counting (TCSPC) accessory. All presented absorption spectra of the ITO/linker/nanoparticle electrodes are obtained under the subtraction of the spectrum of a blank ITO slide from the ones of nanoparticle decorated slides. The non-corrected absorption of a NR decorated substrate and the spectrum of a blank ITO slide are matching well at wavelengths were the particles are not optical absorbing (SI Figure 1).

### Synthesis of CdSe Dots

CdSe dots were synthesized by using a slightly modified procedure developed by Carbone et al. (2007). 0.06 g CdO, 3 g TOPO and 1.7 g ODPA were placed in a 25 mL three-neck flask. The mixture was heated up to 150°C under vacuum and degassed for 1 h. Subsequently, the reaction mixture was heated up to 300°C under an argon atmosphere before 1.8 mL TOP were injected. After a temperature increase to 380°C, which leads to a clear solution, 1.8 mL TOP:Se (0.058 g Se in 1.8 mL TOP) solution were quickly injected. After recovering of the temperature the reaction was quenched with 6 mL ODE and the heating mantel was removed. The reaction mixture cooled down, before 5 mL toluene were added at 70°C and the CdSe dots were precipitated by the addition of 5 mL methanol. All nanoparticles were precipitated via centrifugation for 10 min (3.885 RCF). The precipitate was purified by adding 5 mL of toluene and 5 mL of methanol, subsequent centrifugation for 10 min (3.885 RCF), and dissolution in 5 mL of toluene. This process was repeated one time. The CdSe dots were stored in 6 mL toluene. TEM micrographs of the synthesized CdSe dots were measured (SI Figure 2).

### Synthesis of Dot-in-Rod CdSe/CdS NRs

The CdS shell growth was adapted from Carbone et al. (2007). In a synthesis 0.06 g CdO, 3 g TOPO, 0.16 g ODPA, and 0.08 g HPA were placed in a 25 mL three-neck flask, heated up to 150°C and degassed for 1 h. The temperature was increased to 300°C under an argon atmosphere. After the desired temperature was reached, 1.8 mL TOP were injected followed by an increase of the temperature to 380°C. At this temperature 1.8 mL TOP:S (2.21 mmol) containing 400 μM of CdSe dots [the concentration was determined by UV/Vis spectroscopy and calculation according the work of Yu et al. (2003)] were injected and the reaction mixture was heated for 8 min. After the reaction mixture was cooled down, 5 mL toluene were added at 70°C and the CdSe/CdS NRs were precipitated by the same procedure as the CdSe seeds and stored in 5 mL toluene. TEM images of the synthesized nanorods are displayed (SI Figure 2).

### Pre-silanization Preparation of ITO Slide

The ITO slides were cleaned via a modification of the common known RCA (Radio Corporation of America) cleaning procedure (Kern and Puotinen, 1970). The slides were cut into pieces with the lateral dimensions of 1.5 × 3.0 cm. The slides were placed in a polytetrafluoroethylene (PTFE) holder, which is equipped with a stirring bar. The holder was placed in a beaker. Volume parts of H_2_O_2_ (30%), NH_3_ (28–30%) and H_2_O were mixed in the composition 1:1:5. The ITO slides were completely covered with the solution, which was stirred and heated to a temperature of 70°C for at least 2 h. After the cleaning the slides were rinsed with deionized water and dried under air stream.

### Functionalization of ITO Slides With Linker Molecules

After the cleaning process the cleaned slides were placed in a PTFE holder equipped with a stirring bar and covered with a specific amount of toluene. The system was heated to 60°C and stirred. A volume of the silane linker molecule (1.0 mL MPTMS, 0.2 mL APTMS, 1.5 mL 11-thiol, or 0.3 mL 3-amine) was pipetted into the toluene. After 24 h, the reaction was stopped by removing the slides from the functionalization solution and dipping them into pure toluene to remove residual not bound silane molecules.

### Decoration of ITO/Linker System With CdSe/CdS Nanorods

**Figure 1:**
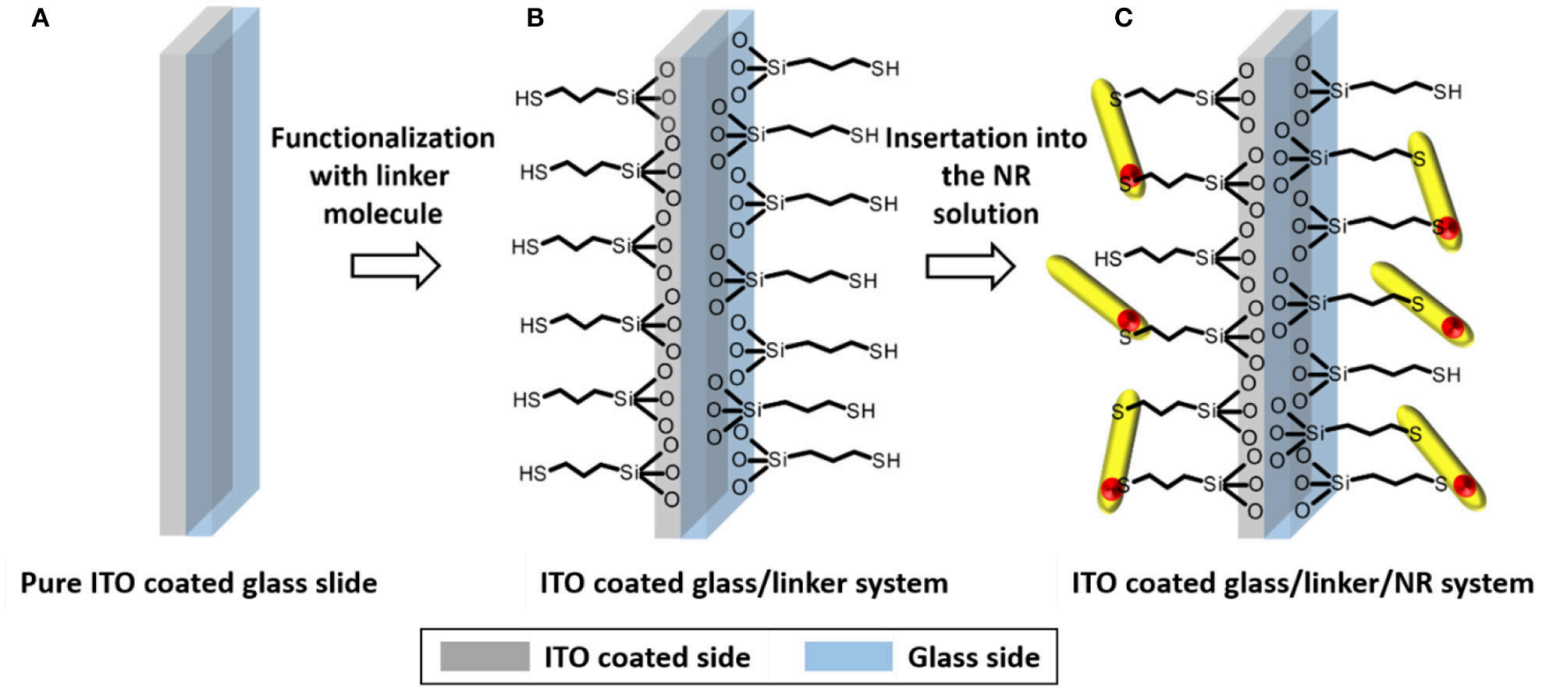
Scheme of the first two steps of substrate preparation. The example of the functionalization of a **(A)** cleaned and surface activated slide **(B)** via MPTMS and the subsequent decoration of the resulting surface via **(C)** CdSe/CdS NR is shown.

The silane functionalized slides were immersed into 10 mL of a solution of CdSe/CdS nanorods in toluene with a Cd-concentration of 15.2 mmol/L. The screw cap vial is placed in a shaker and shacked for 24 h with a velocity of 250 rpm. After this process the functionalization is stopped by removing the ITO slide from the coating solution and immersing it for 2 min in pure toluene. The last step is necessary to remove unbound NRs, which could otherwise form agglomerates on the slide surface during the final drying process. The introduced mechanism is presented as a scheme in Figure 1.

After linking of CdSe/CdS NRs to the slides and a second cleaning step the slides appear slightly yellowish. A comparison of the UV/Vis absorption spectra of pristine nanoparticles in solution and the prepared CdSe/CdS decorated ITO glass slides (under subtraction of the ITO absorption) shows the same optical band edge of CdS at ca. 460 nm, but slight differences at lower wavelengths (SI Figure 3). These differences are probably caused by interferences, which are the result of the glass/ITO/linker/NR layer stacking. The emission maximum of the particles in solution and on the substrate differs by 3 nm, which is probably caused by the appearance of further electronic surface states by linking (SI Figure 4).

### Polishing of ITO Coated Glass/Linker/NR System

**Figure 2:**
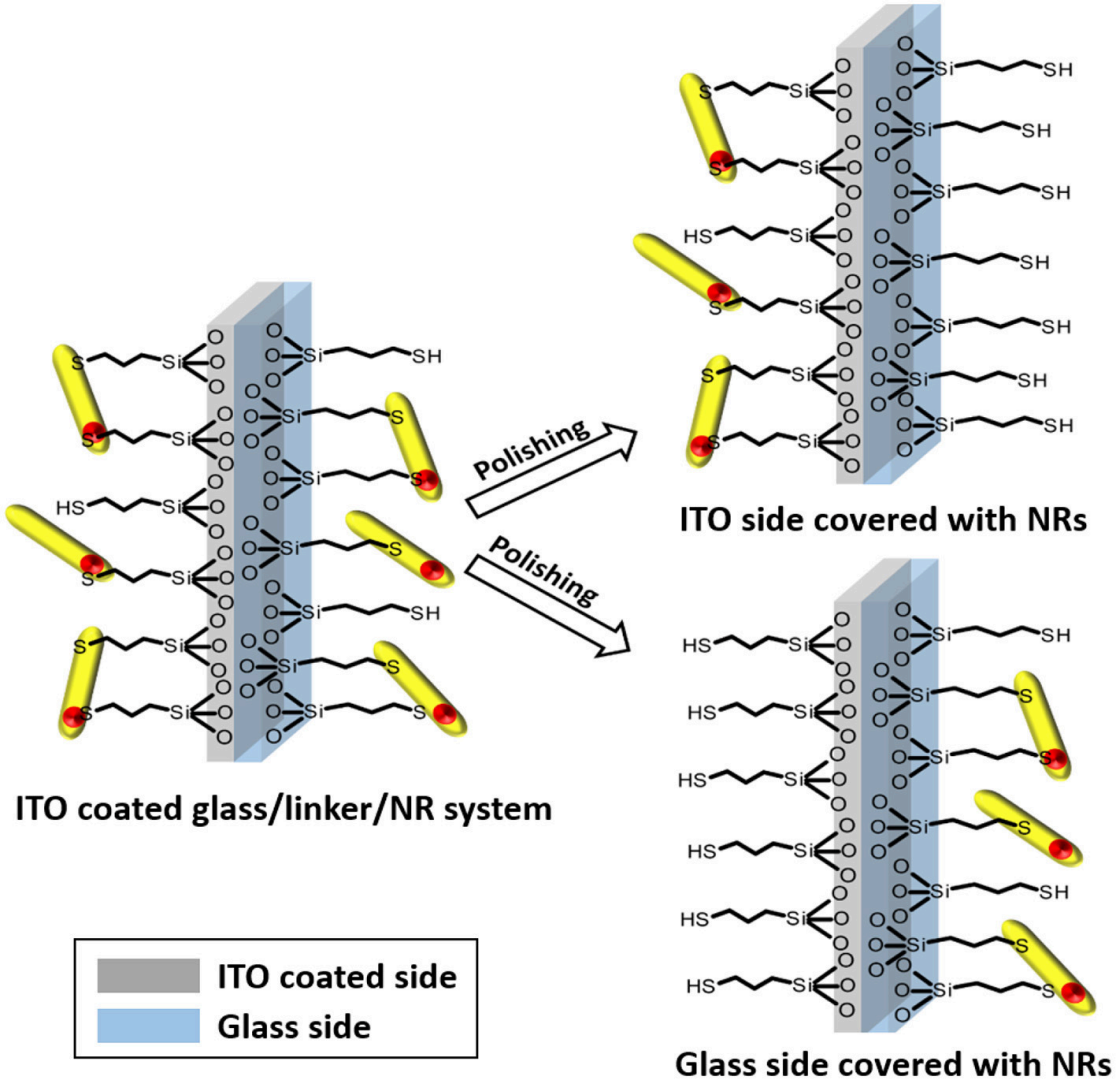
Schematic illustration of the selective polishing of the different sides of the substrate. The result of this process are two comparable systems with completely different electronic interconnections, namely between NR and ITO or NR and glass.

A lint–free paper towel soaked with 2-propanol was applied to polish the desired side of the substrate. Steady wiping for 5 min polished the slide. The effect of polishing is immediately visible under UV-irradiation. The paper towel starts to fluorescence in the same color as the nanoparticles. An overview of the polishing process is delivered in a scheme (Figure 2).

## Results and Discussion

**Figure 3:**
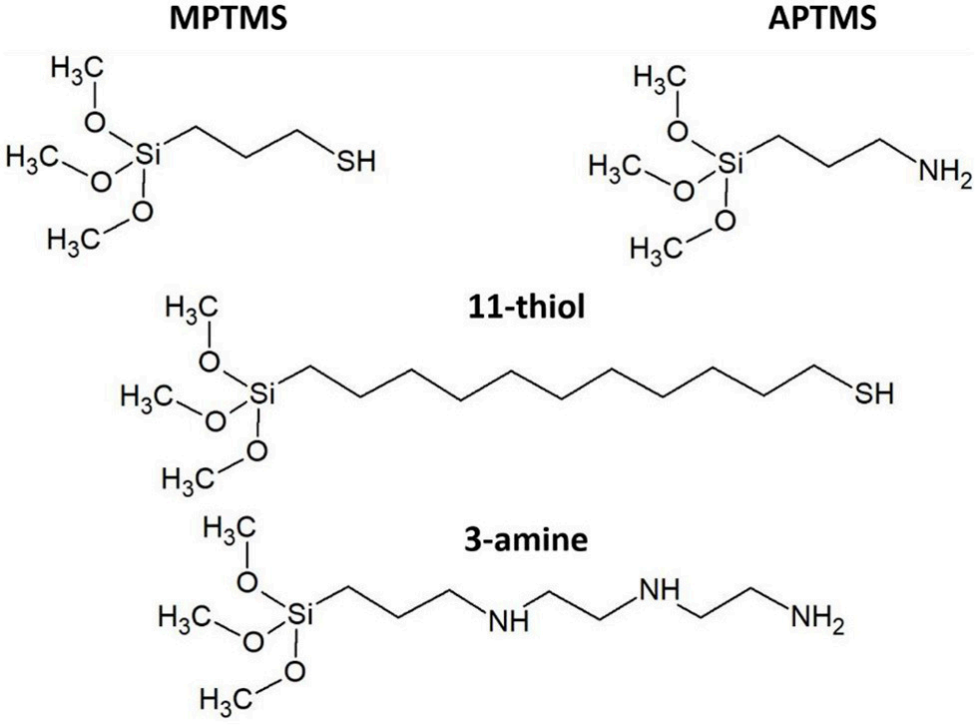
The four different applied silane molecules are based on two thiols (MPTMS and 11-thiol) and two amines (APTMS and 3-amine).

A perfect homogeneous molecular layer of the applied silane molecule (Figure 1) was considered as the target of the silanization reaction. This is elementary for our experiments, since in a monolayer of particles every linked nanoparticle should show similar electronic interconnection with the substrate (Figure 2). The applied thiol and amino silanes are well-known to yield particle monolayers (Brust et al., 1998; Su et al., 2007; Yokota et al., 2012) (Figure 3). Thick multilayers, which are known by literature, would circumvent the required electronic equality of particles connected to the same surface (Wang and Wang, 2004a; Howarter et al., 2006; Kannan and John, 2010). The reaction parameters of the silanization via mercaptosilane and aminosilane linkers have to be modified in different ways, to achieve a monolayer in each case. Aminosilanes form under the same conditions much thicker layers than their thiol counterparts since the amine group is auto catalyzing the hydrolysis of the methoxy groups of the silanes (Sunkara and Cho, 2012). In our experiments the same concentration of aminosilanes and mercaptosilanes in the functionalization step leads to turbid and sometimes opalescent substrates in case of the aminosilanes. The amine based systems showed in this case an inhomogeneous lateral distribution of nanorods, which was even visible by bar eye. Attention was paid to this fact by using a lower concentration of aminosilanes for the functionalization.

The presence of a monolayer was verified by UV/Vis spectroscopy in all cases as demonstrated by us in an earlier work (see SI for details, SI Tables 1, 2). SEM micrographs of a pristine ITO coated glass substrate and an ITO/MPTMS/NR substrate are displaying remarkable differences between the systems. In case of a decorated substrate the rough ITO is covered with small white spots. This spots have an elongated shape and can be identified as nanorods (see SI Figure 5).

**Figure 4:**
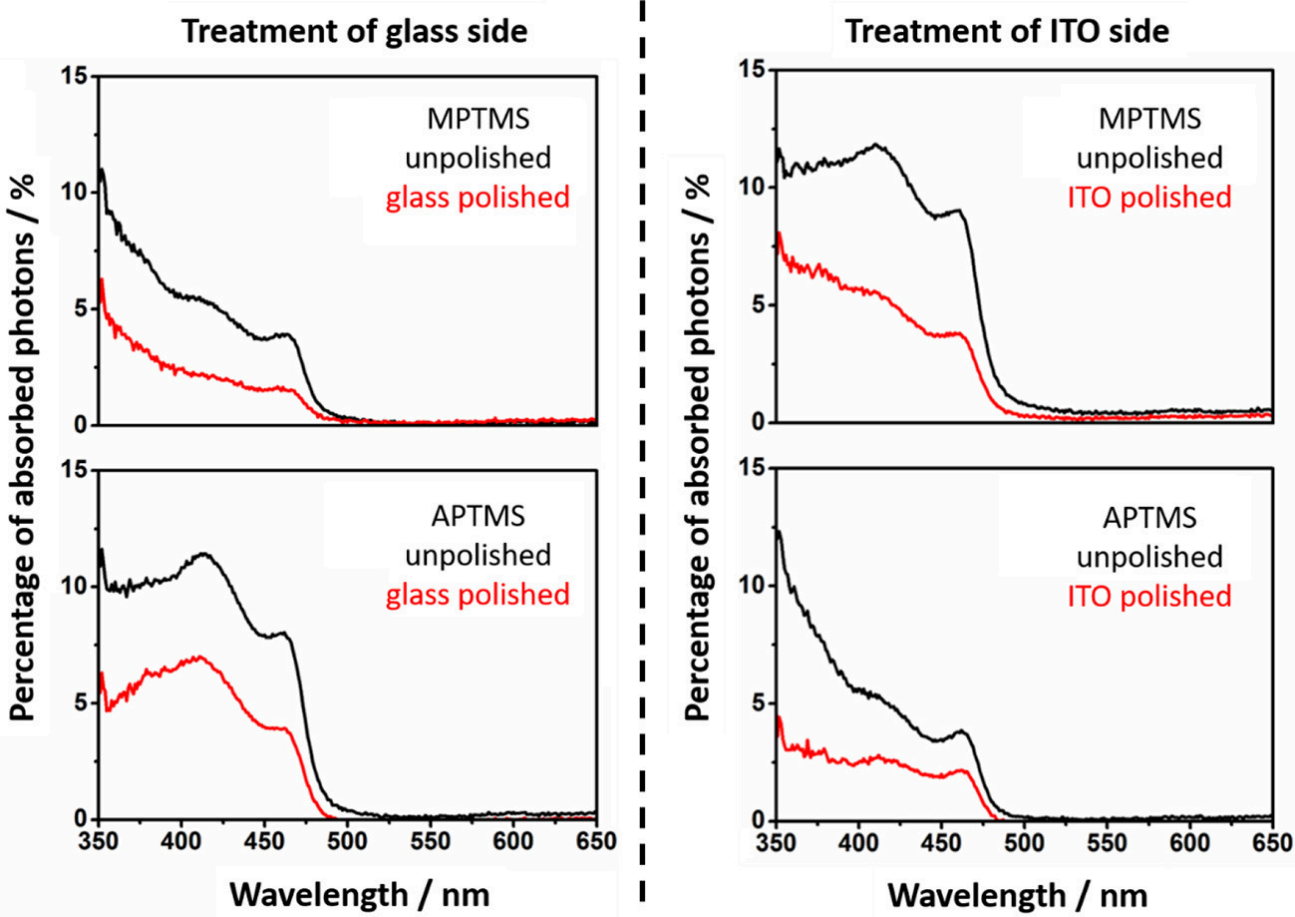
Absorption spectra of the MPTMS and APTMS functionalized substrates with NR decoration. The spectra of unpolished slides are given in black. The absorption spectra of polished slides are red. It is always mentioned if the glass or ITO side is polished. The shape of the NR based absorption differs slightly from sample to sample since interference effects are present.

**Figure 5:**
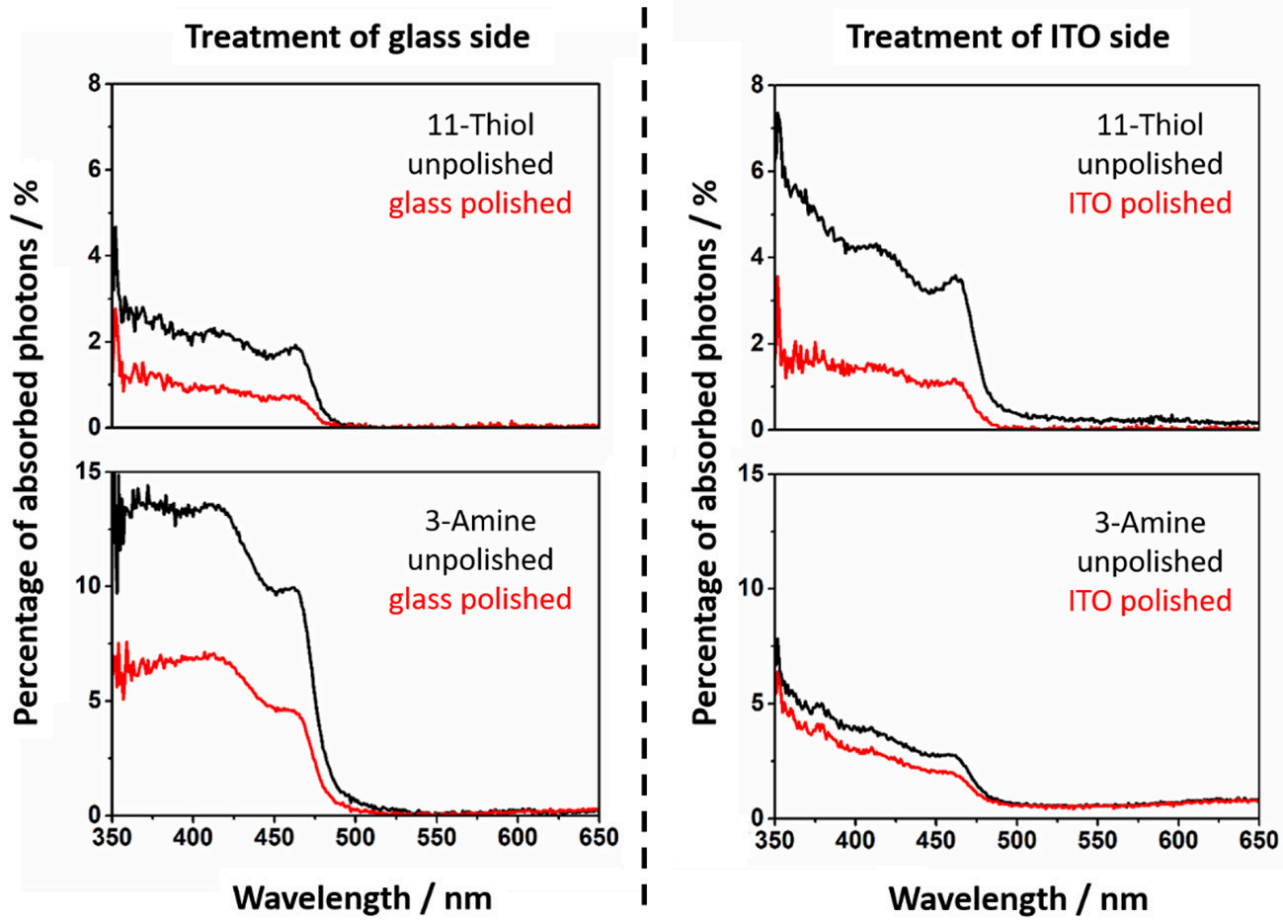
The absorption of NRs at 11-thiol and 3-amine functionalized substrates is shown. The spectra of unpolished slides are presented (black). The absorption of polished slides are red.

By recording absorption spectra before and after polishing the nanorod related percentage of absorbed photons of particle decorated slides can be calculated (Figures 4, 5).

The calculated values of the decorated and undecorated particles differ by a factor of two (Figures 4, 5). The explanation for this observation is the existence of approximately the same degree of coating at both surfaces of the slides, which is one of the fundamentals of the later performed fluorescence analysis of the slides.

The absorption of the NRs at polished substrate is always lower than 5%. This is in fact in good agreement with calculations in our previous work (Miethe et al., 2018). This calculations lead to the interpretation that the optical density is caused by a nanorod coverage in the monolayer regime (Miethe et al., 2018). The similar chemical behavior of both sides of the slide suggests a similar degree of functionalization of the soda lime glass and the ITO. The same behavior is found for every of the four applied linkers (Figure 3).

**Figure 6:**
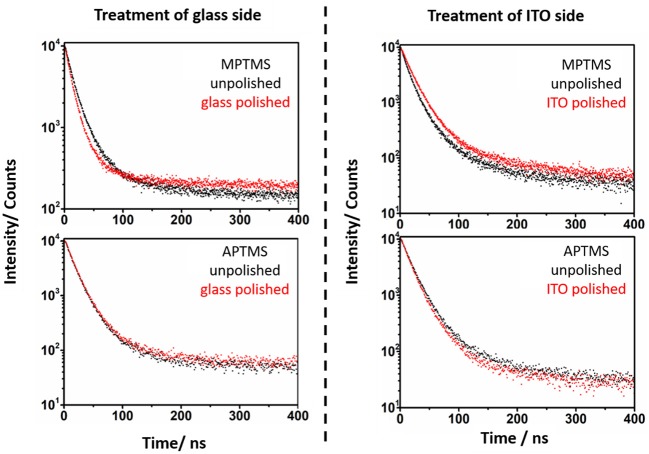
Photoluminescence lifetime spectra of the MPTMS and APTMS based systems before (black) and after polishing (red). The specific side, which is polished is mentioned in the spectra. It is obvious, that the polishing of the glass side of MPTMS based systems decreases the PL lifetime. In contrast to this, a polishing of the ITO side increases the PL lifetime.

**Figure 7:**
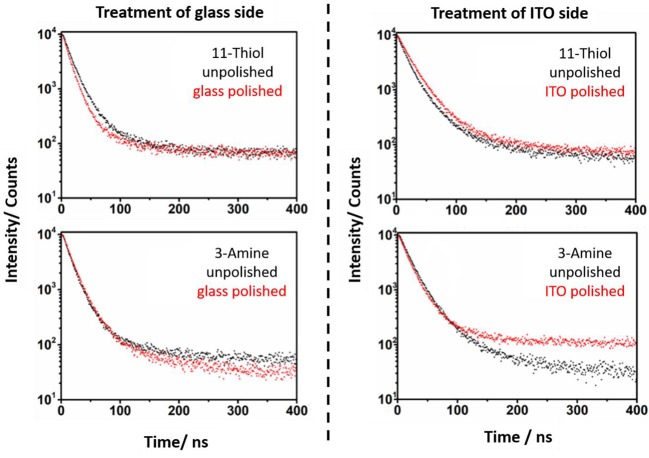
The PL lifetime of NR, which are linked to substrates via 11-thiol and 3-amine, is measured. The spectra of unpolished (black) and polished (red) slides is shown. The lifetimes of unpolished and polished slides vary strongly in case of 11-thiol but not in their 3-amine counterparts.

Photoluminescence spectra of the pristine NRs in solution as well as of the slides show a PL at 612 nm (SI Figure 4). A simple comparison of PL lifetime measurements of samples with one polished ITO or glass side with the spectrum of exactly the same slide before polishing shows a remarkable difference in the decay signal. In case of the thiol based linkers MPTMS and 11-thiol a shorter lifetime for NRs connected to ITO can be found in relation to unpolished systems (Figures 6, 7), whereas the glass connected NRs exhibit a higher PL lifetime compared to the unpolished system. More details of the PL lieftime measurements are given in the supporting information of this paper.

In case of amine linked CdSe/CdS particles it was more challenging to find any difference between the PL decay curves of the glass and ITO connected particles (Figures 6, 7). Here, the effect of polishing on the glass and ITO connected particles is small.

This major observation was at least three times repeated with three samples for every unpolished, glass polished and ITO polished substrate functionalized with one of the four utilized linker molecules. This leads to a data collection, which provides a standard deviation in every measurement point. The basis of the following kinetical PL description of samples is given by the simple determination of the kinetic constant of fluorescence quenching processes in NRs in solution *k*_s_. This kinetic constant is derived from equation 1.

(1) $$\begin{array}{r} I=I_{mono}\cdot e^{-k_{s}t} \end{array}$$

The pristine tri-*n*-octylphosphine and tri-*n*-octylphosphine oxid covered CdSe/CdS NRs in toluene solution exhibit a PL lifetime of 24 ns and decay constant *k*_*s*_, which is caused by the sum of all non-radiative and radiative processes of 41•10^6^ s^−1^.

**Figure 8:**
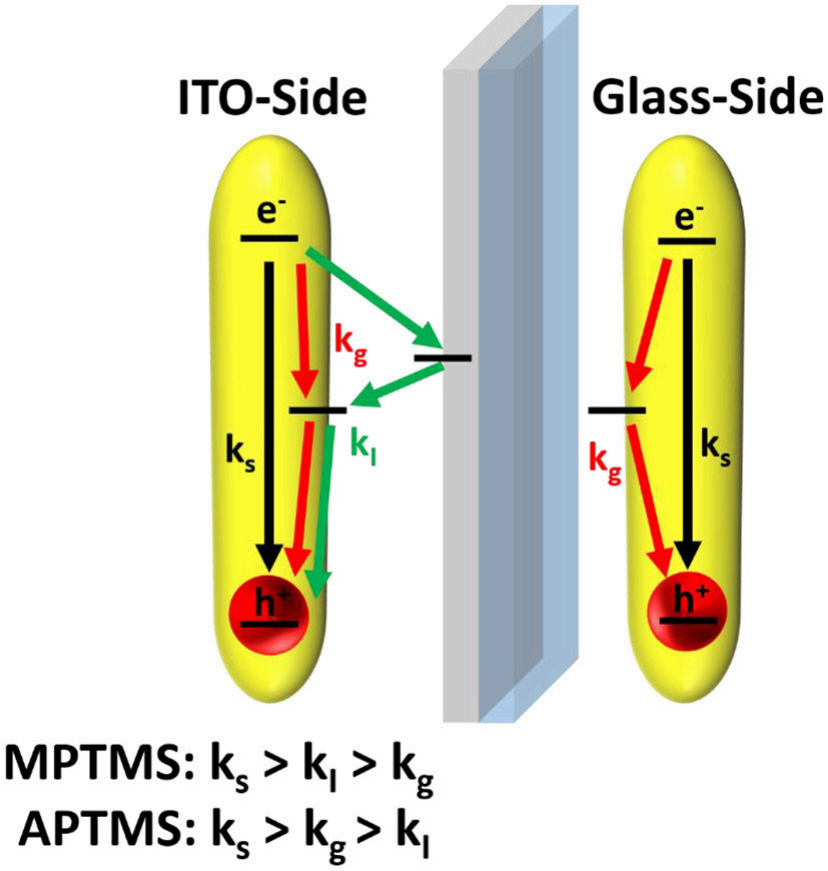
A scheme of the described exciton quenching is based on three different mechanisms. The phenomenological kinetic constant *k*_s_ describes all quenching mechanisms, which are existing in case of NRs in free solutions. The by linking molecules introduced trap state related quenching processes are described by the kinetic constant k_g_. Only excitons of NRs linked to the ITO side of the slide can undergo a quenching process, which is related to an interconnection between them and the free charge carriers of ITO (accounted for by the kinetic constant *k*_I_).

PL lifetimes of the NR covered substrates were obtained via mono or biexponential fitting. Particle monolayers with homogenous nanoparticle-surface connectivity should be describable via a monoexponential decay function. This function (Equation 2 and Equation 3) was applied to describe the additional kinetic influence of trap states created by linking *k*_g_ in case of every polished sample. Whereby only the NRs linked to ITO develop additional ITO related quenching kinetics *k*_I_. The existing kinetic mechanisms are described in Figure 8.

(2) $$\begin{array}{r} I=I_{mono}\cdot e^{-\left(k_{s}+k_{g}\right)t} \end{array}$$

(3) $$\begin{array}{r} I=I_{mono}\cdot e^{-\left(k_{s}+k_{g}+k_{I}\right)t} \end{array}$$

PL decays of unpolished NR decorated ITO slides (Figures 6, 7) were fitted with a biexponential decay function (Equation 4). This fitting protocol is justifiable by the circumstance that two ensembles of emitting nanoparticles exist on an unpolished slide. One at each side.

(4) $$\begin{array}{r} I=I_{bi}\cdot e^{-\left(k_{s}{+k}_{g}\right)t}+I_{bi}\cdot e^{-\left(k_{s}{+k}_{g}+k_{I}\right)t} \end{array}$$

Since the contribution to the total signal of every side to the system should be 50% the factor *I*_bi_ was assumed to be only half of the factor *I*_mono_ for every term of the biexponential fit (Equation 5).

(5) $$\begin{array}{r} I_{mono}=2{\cdot I}_{bi} \end{array}$$

An overview of the values of the kinetic constant of the non-radiative decay components is given in Table 1. An overview of the PL lifetime of the measured samples, which are obtained by mono and biexponential fits is shown in the supporting information (SI Figure 6 and SI Table 3).

**Table 1:** The calculated kinetic constants of slide/linker/NR system are shown for the four applied linkers.

	**Kinetic Constant/10^6^s^−1^**			
	**Polished system**		**Unpolished system**	
	**Monoexponential fit**		**Biexponential fit**	
**Applied linker**	**k**_**I**_	**k**_**g**_	**k**_**I**_	**k**_**g**_
MPTMS	24 ± 5	4 ± 2	39 ± 8	1 ± 6
APTMS	3 ± 3	17 ± 3	4 ± 6	17 ± 14
11-thiol	44 ± 2	2 ± 1	38 ± 31	1 ± 10
3-amine	7 ± 7	20 ± 10	3 ± 20	24 ± 7

*The values of unpolished systems were derived from biexponential fits. The values of k_g_ are derived from monoexponential fits of ITO polished samples. Monoexponential fits of glass polished samples lead under consideration of K_g_ to k_I_*.

The kinetic constant *k*_g_ is only relevant in case of aminosilane linkers (Table 1). NRs that are connected by mercaptosilane linkers to ITO are the only system, which show an ITO related quenching process. This process is described by the constant *k*_*I*_. The biexponential fit of unpolished slides is required to verify the kinetic constants (Equation 4 and Table 1). In case of polished slides, the same kind of constant is derived from monoexponential fits (Equation 2 and 3, Table 1). Note that the standard deviation of the kinetic constants calculated in two different ways at glass and ITO are confirming the validity of our approach. This is a good indication that the measured biexponetial behavior of the unpolished samples is indeed a simple superposition of the two sides of the sample.

The simple linking of thiol groups to the particle surface does not change their PL kinetics. This is proven by the same PL lifetimes for unlinked CdSe/CdS NRs and NRs linked to the glass side (SI Figure 6 and SI Table 3).

Thus, the obtained results show that the lifetime of nanorods decreases more by a chemisorption of amine groups than by thiol groups. Nearly no noticeable decrease of the PL lifetime is recognizable when NRs are connected via aminosilanes to the conductive ITO side of the slide. This finding can be explained either by a much weaker interaction between ITO and nanoparticles when amine linkers are employed, or possibly also by a thicker layer of aminosilane formed. The latter effect is less likely since much lower amounts of aminosilanes were employed during functionalization. A connection between particle and slide via mercaptosilanes instead leads to a strong decay via an additional particle-ITO interaction (represented by *k*_I_).

One of the major questions, which arises by studying this spectroscopic phenomenon, is how ITO can shorten the PL kinetics and why the effects depends more on the functional group of the linker than its length. The literature gives answers to this question. The Fermi energy of ITO has a potential, which is placed in the band gap of the semiconductor (Tang et al., 2007; Zillner et al., 2012). An arrangement of energy levels like this and the multiple times higher amount of free charge carriers in ITO than in the nanoparticles lead to an injection of electrons from the ITO into unoccupied electronic states in the energy levels of the photoexcited metal chalcogenide semiconductor. The injection of electrons is responsible for the faster quenching of holes obtained by photoexcitation of the metal chalcogenide particles. Holes are often trapped at subvalent sulfur atoms at the particle surface. The amount of the trapped charge on nanoparticles of metal chalcogenides is investigated by Fengler et al. (2013). The whole charge transport is describable by the kinetic constant *k*_I_.

It is assumed in the work of Grandhi et al. that the chemisorption of amines at the NR surface leads to a quenching of hole traps at the NR (Grandhi et al., 2016). This would also lead to a short PL lifetime, which can be verified by the obtained measurements in Table 1. On the other hand, there are no holes available for quenching by injection from ITO. Under these conditions there seems to be no influence of ITO on the PL kinetics (Cooper et al., 2011; Zeng and Kelley, 2016). This mechanism can be described by the kinetic constant *k*_g_.

In summary both types of linkers enable a quenching of PL kinetics. The aminosilane linker under direct hole quenching and the mercaptosilane linker via indirect charge injection from ITO. Literature shows that the described PL influencing processes are also strongly interconnected to the population of electronic states in the slide (Wehrenberg and Guyot-Sionnest, 2003; Gooding et al., 2008; Li et al., 2017).

## Conclusion

In the presented work, we showed an experimental setup, which relays on the comparison of classical prepared CdSe/CdS nanorod decorated ITO coated glass substrates with unusual reference samples. The samples consist of a slide at which a soda lime glass side and a ITO coated side are covered with the same particles. The absorption spectroscopy of the products proves that all four applied silane linker molecules (APTMS, MPTMS, 11-thiol, 3-amine) are applicable to form monolayers of particles. Even though the silanization behavior of the linker molecules is extremely different, all of them have in common that they show no specific difference in affinity to the soda lime or the ITO side of the glass substrate. This is the fundamental experimental condition to describe the PL decay with a biexponential fit representing the two different species in the system. The evaluation of the differences between the PL decay of pristine NRs, glass connected and ITO connected NRs yields kinetic constants, which describe the influence of the electronic coupling in the system. The way of interaction of ITO with particles is clearly depending on the functional group of the applied linker molecule. Mercaptosilanes are forming hole trap states at the surface of the NRs, which enable the injection of electrons from the ITO and by this a faster PL quenching mechanism. On the other hand, there seems to be only a small influence of this trap states on the PL in the absence of ITO. In case of aminosilanes functionalized substrates, a strong decrease of the PL lifetime of the connected NRs can be observed. The electronic interconnection of ITO and NRs, which is initialized via APTMS and related to the decrease of the PL lifetime however is very weak. The PL quenching effects and their linker related differences can be understood as a possibility to use the experimental setup reported in our work to create approaches for an analytical differentiation between various linker molecules.

The collected results lead to the conclusion that the electronic connection of photoluminescent nanoparticles to the electronic states of the ITO can be proven via contact free measurements of the PL lifetime of the particles. Since there is a growing interest in ITO surfaces for sensing applications and the possibility to utilize specific linker molecules as sensor interface, PL lifetime measurements could be applied as a productive tool of analysis.

The presented work might be the basis for more advanced investigations, which could reveal the influence of the semiconductor heterostructure and by association the effect of the occupation of electronic states in transparent conductive oxides. Even the influence of both kinds of investigated molecules in mixed linker systems could be interesting.

In summary there should be strong motivation to consider the obtained mechanisms of charge transport in case of applications like photoelectrochemical sensing and energy harvesting in order to amplify the desired performance.

## Author Contributions

JM has conducted all electrochemical measurements and has drafted the manuscript. FL has synthesized the investigated nanocrystals. DD and NB have supervised the project and were involved in all data interpretation as well as in writing and correcting the manuscript.

### Conflict of Interest Statement

The authors declare that the research was conducted in the absence of any commercial or financial relationships that could be construed as a potential conflict of interest.
