# Supplementary material for: Photoluminescence Lifetime Based Investigations of Linker Mediated Electronic Connectivity Between Substrate and Nanoparticle
Source: Front Chem. 2019 Apr 10;7:207. doi: 10.3389/fchem.2019.00207 (PMC6467932; doi:10.3389/fchem.2019.00207)
Supplement: Supplementary file 1 [file Data_Sheet_1.pdf]

# Supporting Info

## Fluorescence Lifetime based Investigations of Linker Mediated Electronic Connectivity Between Substrate and Nanoparticle

Jan F. Miethe, Franziska Lübkemann, Nadja C. Bigall, Dirk Dorfs*

*Institute of Physical Chemistry and Electrochemistry, Leibniz Universität Hannover, Callinstr. 3a, 30167 Hannover, Germany*

*dirk.dorfs@pci.uni-hannover.de

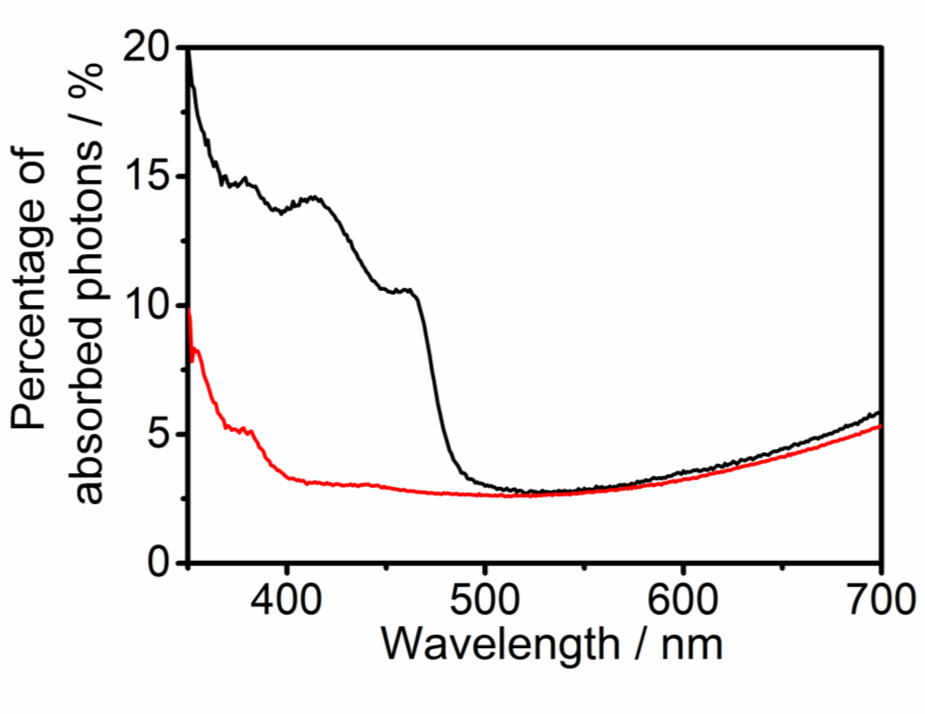


**SI Figure 1** The absorption spectra of a ITO/MPTMS/NR system (black) and a blank ITO electrode (red) are compared to each other.

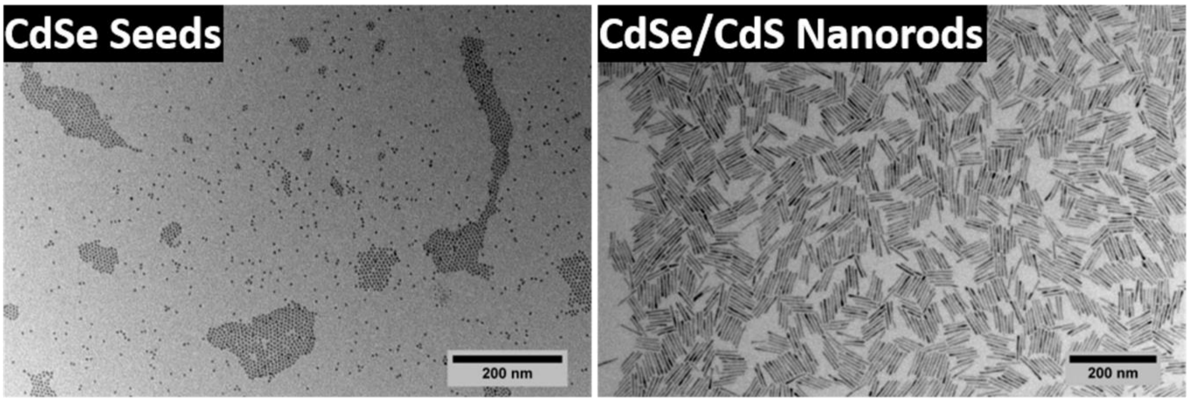


**SI Figure 2** TEM micrographs of CdSe dots (left) and the CdSe/CdS nanorods (right). The uniform size distribution of the nanoparticles is visible.

## Calculations of the occupied surface area

Our mathematical prove of a surface occupation, which is in the monolayer regime, is based on the absorption of the nanoparticles. The applied model is based on the assumption that the shape of the nanorods is estimated to be a perfect cylinder, the particles are only made out of CdS and the nanorods are lying flat at a flat substrate surface. The last approximation is clearly not the case since ITO shows by product a root-mean-squared surface roughness of 1 to 2nm.

UV/Vis spectroscopy was applied to determine the extinction coefficient with a cuvette of 1cm pathway (Table SI-1).

**SI Table 1** The concentration of cadmium was determined in case of the synthesis solution via atom absorption spectroscopy. The concentration of the diluted synthesis solution is via UV/Vis measurement related to an absorption to gain an extinction coefficient.

| Sample | Concentration of Cd ($mol/cm^3$) | Absorption (%) | Extinction Coefficient ($cm^2/mol$) |
|---|---|---|---|
| **Synthesis Solution** | $2.84\cdot10^{-4}$ | - | - |
| **1 µL of the Synthesis Solution in 3000 µL of Toluene** | $9.47\cdot10^{-8}$ | 12.7 | $6.23\cdot10^{5}$ |

The absorption is directly proportional to the number of nanorods in the cross section of the optical path of the UV/Vis measurement. An extinction coefficient is required to calculate the absolute number of particles. The amount of cadmium or cadmium sulfide can be calculated. This makes it possible to connect a cadmium concentration to a number of nanorods.

The maximum area, which can be occupied by a nanorod with a diameter of 3 nm, is $1.2 \cdot 10^{-12}$ cm$^2$. Under consideration of the number of particles per area and the occupied area per nanorod the percentage of the surface, which is covered by particles can be calculated.

The volume of a nanorod $V_{rod}$ can be calculated under consideration of the length $l$ and radius $r$ of the rods (equation 1).

$$V_{rod} = \pi r^2 l \qquad (1)$$

The absolute number of nanorods is calculated under consideration of the density $\rho_{CdS}$ and molar mass $M_{CdS}$ of CdS (equation 2).

$$n_{CdS} = \frac{V_{rod} \cdot \rho_{CdS}}{M_{CdS}} \qquad (2)$$

**SI Table 2** Calculation of the surface concentration of material and by this the absolute number of nanorods from the absorption value are shown. The percentage of surface, which is covered by particles can be assumed in the end.

| Sample | Absorption (%) | Concentration of Cd (mol/cm²) | Number of Nanorods (1/cm²) | Percentage of Occupied Area (%) |
|---|---|---|---|---|
| Theoretical volume with the dimension of 1 cm³ | 12.7 | $9.47 \cdot 10^{-8}$ | $9.41 \cdot 10^{12}$ | 1130 |
| MPTMS glass polished | 1.5 | $1.11 \cdot 10^{-8}$ | $1.11 \cdot 10^{12}$ | 133 |
| MPTMS ITO polished | 3.1 | $2.32 \cdot 10^{-8}$ | $2.3 \cdot 10^{12}$ | 277 |
| APTMS glass polished | 3.3 | $2.45 \cdot 10^{-8}$ | $2.43 \cdot 10^{12}$ | 292 |
| APTMS ITO polished | 1.8 | $1.34 \cdot 10^{-8}$ | $1.33 \cdot 10^{12}$ | 160 |
| 11-thiol glass polished | 0.6 | $4.64 \cdot 10^{-9}$ | $4.61 \cdot 10^{11}$ | 55 |
| 11-thiol ITO polished | 0.9 | $6.90 \cdot 10^{-9}$ | $6.86 \cdot 10^{11}$ | 82 |
| 3-amin glass polished | 4.1 | $3.04 \cdot 10^{-8}$ | $3.02 \cdot 10^{12}$ | 363 |
| 3-amin ITO polished | 2.3 | $1.70 \cdot 10^{-8}$ | $1.69 \cdot 10^{11}$ | 203 |

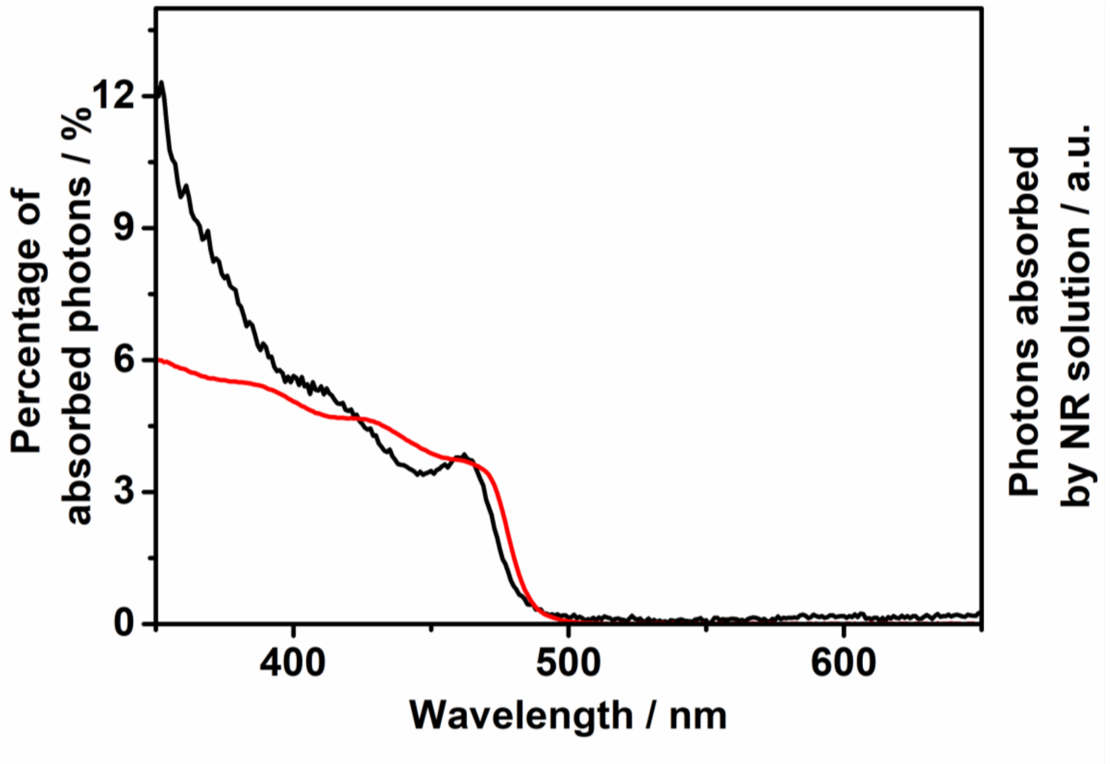


**SI Figure 3** The absorption spectra of the system ITO/APTMS/NR functionalized electrode (black) and a toluene based solution of the same particles (red) are compared. Differences of

the absorption are caused by interferences, which become very strong at the point were also the ITO starts to absorb.

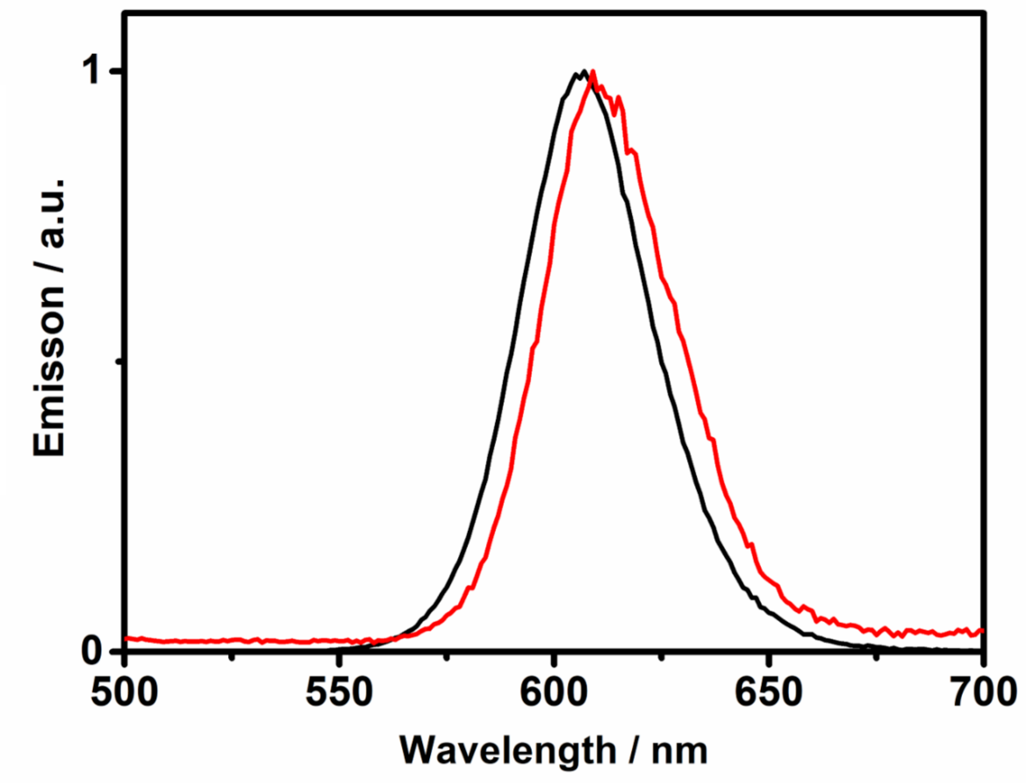


**SI Figure 4** The photoluminescence of a ITO/APTMS/NR (red) and the same particles in toluene solution (black).

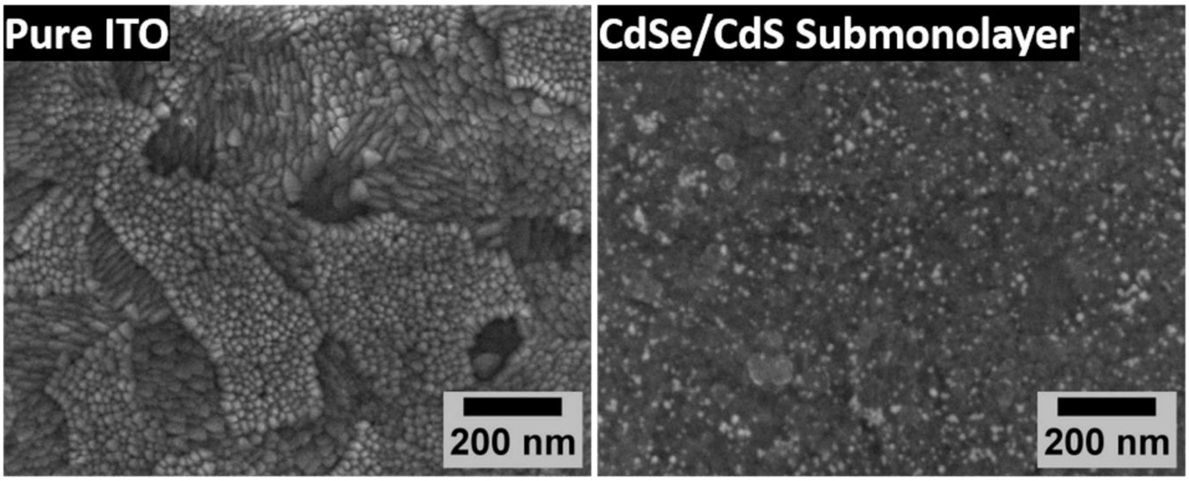


**SI Figure 5** The rough surface of pure ITO presents crystalline domains. The nanorods at a typical decorated substrate are visible in form of small, bright, and elongated dots.

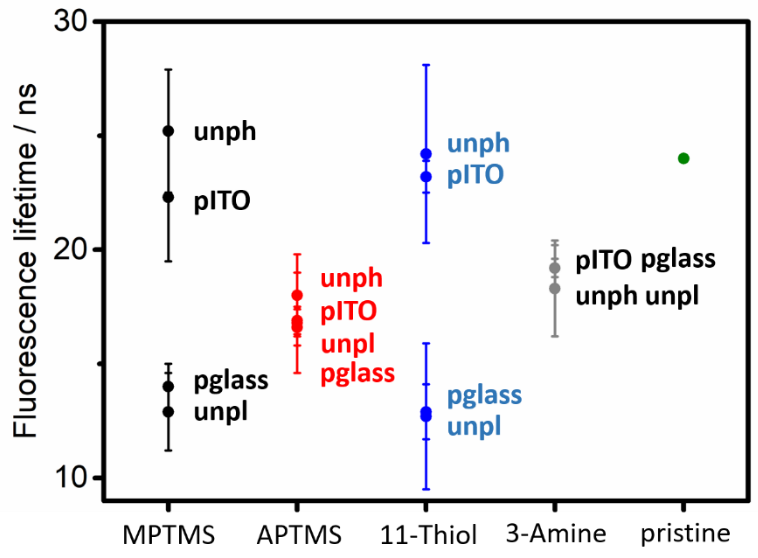


**SI Figure 6** The PL lifetimes of systems, which are based on the four different linkers MPTMS (black), APTMS (red), 11-thiol (blue) and 3-amine (grey) are shown. The lifetime of pristine NRs in toluene solution (green) is given as a reference value. There are four states of every linker system, which are considered during data collection. Values obtained from the biexponential fit of the PL liftime decay of unpolished systems are separated into the higher (unph) and lower (unpl) value. The polished systems are separated by factor if the glass side (pglass) or its ITO counterpart (pITO) is polished. Since every value was measured under consideration of at least three different electrodes of the same type a calculation of the standard deviation was possible. Standard deviations of the lifetimes values are given as bars.

**SI Table 3** The measured values of electrode/linker/NR system based on different linkers are shown for the four applied linkers. The values of the unpolished systems were obtained under application of biexponantial fits, whereas the polished systems were fitted monoexponantialy. The standard deviation of the samples of every investigated system is given.

| | PL lifetime / ns | | | |
|---|---|---|---|---|
| | unpolished | | polished | |
| **Name of linker** | **Higher lifetime** | **Shorter lifetime** | **NRs at ITO** | **NRs at glass** |
| **MPTMS** | 25.2 ±2.7 | 12.9 ±1.7 | 14.0 ±1.0 | 22.3 ±2.8 |
| **APTMS** | 18.0 ±1.8 | 16.8 ±2.2 | 16.6 ±0.8 | 16.9 ±0.6 |
| **11-thiol** | 24.2 ±3.9 | 12.7 ±3.2 | 12.9 ±1.2 | 23.2 ±0.7 |
| **3-amine** | 18.3 ±2.1 | 18.3 ±2.1 | 19.2 ±1.0 | 19.2 ±0.4 |

**Remarks to the Measurement of the Photoluminescence Lifetime**

The measurements of the photoluminescence lifetime were repeated with three different samples at least for every type of unpolished, glass polished and ITO polished electrode functionalized with one of the four utilized linker molecules. This leads to a data collection, which provides a standard deviation in every measurement point.

The data points of the fluorescence lifetime measurements were fitted with mono- or biexponential fits.

All fits were fitted in the time range of the first 200 ns of the experiment. Since this time scale is one magnitude higher than the highest lifetime measured it is assumed that in good approximation all valuable data points are part of it (Table 1). The dark counts play an inferior role in this time regime and are in good approximation neglected in our fits. Under careful examination of the shown PL kinetic measurements (Figure 6 and 7) it can be seen that the altitude of dark counts differs between the samples. The measurement time to reach the considered amount of PL counts is higher in systems, which contain a smaller amount of photoluminescent particles. In case of this systems the number of collected dark counts is higher in relation to the collected PL counts.
